# Acute Hepatitis of Unknown Origin in Pediatric Age Group: Recent Outbreaks and Approach to Management

**DOI:** 10.3390/jcm12010009

**Published:** 2022-12-20

**Authors:** Neil Patel, Yashendra Sethi, Nirja Kaka, Oroshay Kaiwan, Ishita Gupta, Rahma Sameh Shaheen, Shady Sapoor, Hitesh Chopra, Mihaela Simona Popoviciu, Talha Bin Emran, Simona Cavalu

**Affiliations:** 1 PearResearch, Dehradun 248001, India; 2 Department of Medicine, GMERS Medical College, Himmatnagar 383001, India; 3 Department of Medicine, Government Doon Medical College, HNB Uttarakhand Medical Education University, Dehradun 248001, India; 4 Department of Medicine, Northeast Ohio Medical University, Rootstown, OH 44272, USA; 5 Department of Medicine, Dr. Rajendra Prasad Government Medical College, Tanda 176001, India; 6 Faculty of Medicine, Benha University, Banha 6470031, Egypt; 7 Department of Pharmacy, Chitkara College of Pharmacy, Chitkara University, Punjab 140401, India; 8 Faculty of Medicine and Pharmacy, University of Oradea, P-ta 1 Decembrie 10, 410087 Oradea, Romania; 9 Department of Pharmacy, BGC Trust University Bangladesh, Chittagong 4381, Bangladesh; 10 Department of Pharmacy, Faculty of Allied Health Sciences, Daffodil International University, Dhaka 1207, Bangladesh

**Keywords:** non-HepA–E hepatitis, hepatitis of unknown origin, acute hepatitis, pediatric hepatitis, hepatitis outbreak, SARS-CoV-2 hepatitis

## Abstract

Acute hepatitis has always been a public health concern, but the recent clustering of cases in various parts of the world has drawn some special attention. The sudden rise in cases has mainly been among the pediatric population of around 35 countries around the world, including developed countries such as the United States, the United Kingdom, and European countries. The outbreaks have had a devastating impact, with around 10% of the affected patients developing liver failure. The clinical presentation of patients resembles any other case of acute hepatitis, with the major symptoms being: jaundice (68.8%), vomiting (57.6%), and gastrointestinal symptoms such as abdominal pain (36.1%) and nausea (25.7%). Interestingly, the cases have tested negative for hepatotropic viruses Hep A, B, C, and E, thus giving rise to the terms Hepatitis of Unknown Origin or non-HepA–E hepatitis. Many causes have been attributed to the disease, with major evidence seen for adenovirus and SARS-CoV-2. International agencies have stressed on establishing diagnostic and management protocols to limit these outbreaks. As the understanding has evolved over time, diagnostic and management faculties have found more shape. The current review was designed to comprehensively compile all existing data and whittle it down to evidence-based conclusions to help clinicians.

## 1. Introduction

Viruses are one of the major causes of hepatitis. Hepatitis viruses A, B, and C are the most common causes of viral hepatitis in the United States [1]. Recently, there has been an increase in the number of hepatitis cases in children worldwide. However, a lot of these patients test negative for the most common hepatotropic viruses, A, B, C, and E [2,3,4]. The first public health alert was raised to Public Health Scotland (PHS) on 31 March 2022, with about five children presenting to the Royal Hospital for Children, Glasgow, who were all admitted within a 3-week period for severe acute hepatitis of unknown origin [4]. On further evaluation of the clinical cases, PHS noted that the number of cases of hepatitis since January 2022 was significantly higher than that of the previous years and almost all patients were children under the age of 10 years. At the time, a confirmed case was anyone 10 years or younger who presented since 1 January 2022 with aspartate transaminase (AST) or ALT greater than 500 IU/L [4]. The World Health Organization (WHO) published its first report on this subject on 15th April 2022—“Acute hepatitis of unknown etiology—the United Kingdom of Great Britain and Northern Ireland”, wherein they also noted that three confirmed cases from Spain and five possible and confirmed cases from Ireland had been identified [5]. In the same report, the WHO also highlighted the importance of identifying these cases [6].

Currently, 35 countries have reported 1010 probable cases of acute hepatitis of unknown origin in children with 48% (484) of the cases being reported from Europe and 27% (272) of the cases from the United Kingdom [5]. The Eastern Mediterranean region currently region has the fewest number of cases, with two cases reported to the WHO. In total, 358 cases have been identified in the US from 43 jurisdictions [7]. The median age reported in the US is 2 years and, in the UK, is 3 years [7,8]. Worldwide, the WHO reported that as of 8th July 2022, the majority of the cases were under six years of age [5]. Most of the statistics show a slight female predominance with 48% of patients being males and 52% being females [5,7,8]. According to the CDC, 38% of patients in the US had associated comorbidities [7]. As per the latest report published by the European CDC on 26th August 2022, 89 patients out of 321 (with available information) were admitted to the intensive care unit [3]. In total, 5% of children (46) required liver transplantation with 2% (22) deaths worldwide [9]. Out of these, 13 deaths have been reported in the US and 3 deaths in Europe [3,7]. No deaths have been reported in the UK [10]. Brazil also reported 7 deaths as of 13 June 2022 [11].

Although the most recent report by WHO, dated 12 July 2022, does not define a confirmed case, a working definition of a probable case involves any person 16 years or younger presenting since 1 October 2021 with acute hepatitis (non-hepatitis A–E) and AST or ALT greater than 500 IU/L. A pending case has been defined as any case where the hepatitis A–E serology is pending. WHO also advises against testing for the delta virus in all cases, as it should only be done in HBsAg positive patients [5].

Adenovirus and SARS-CoV-2 virus are the two hypothesized causes. One retrospective study involving 44 children in a single center in the UK found that 90% of children who underwent testing were positive for human adenovirus [12]. Both the CDC and WHO consider these two possibilities, as in Europe, adenovirus has been detected in 52% of cases and SARS-CoV-2 in 16% of cases [5,13]. In the US, 49% of cases tested for adenovirus were positive and 10% of persons under investigation were positive for SARS-CoV-2 [7]. However, there is still limited data for confirmation. COVID-19 vaccination has not been shown to be linked to hepatitis of unknown origin [14,15]. However, this could be due to the fact that children, especially those under the age of 5 years, would not have received the vaccination and this hepatitis has been seen to mainly affect children [15]. Common symptoms that have been reported are nausea or vomiting, jaundice, general weakness, and abdominal pain, with nausea/vomiting being the most common (60% cases) [4,5]. Abdominal pain and diarrhea are the main symptoms that have led to hospitalization [14]. One case of encephalopathy has also been documented [2].

Several guidelines currently recommend testing for hepatitis A–E to rule those out first and then continue testing for other viruses, especially adenoviruses (via blood specimen, respiratory specimen, and stool specimens, whenever required) and SARS-CoV-2 virus (serology testing by detecting antibodies against the virus) [10,16,17]. PAHO also recommends testing for heterotopic diseases endemic to particular areas such as malaria [16]. Supportive treatment is currently the mainstay [10]. The WHO and CDC have released guidelines and instructions for parents as well as providers [5,17,18,19].

**Figure 1:**
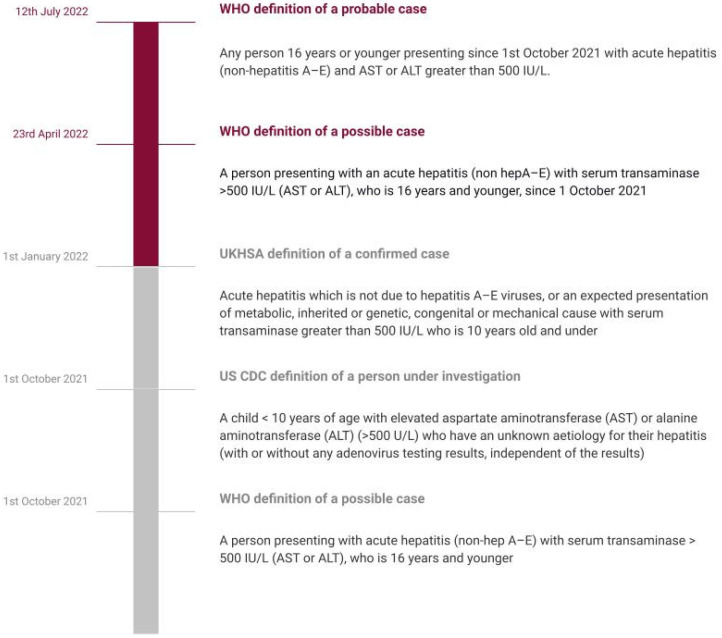
Timeline showing evolving definitions.

During the existing COVID-19 pandemic, this outbreak of hepatitis cases needs to be dealt with urgently. Given the severity and existing gaps in our understanding of this current public health crisis, we reviewed the current and latest literature to provide a detailed overview of key features associated with hepatitis of unknown origin. Our review aims to summarize the current understanding of etiopathogenesis, clinical and socio-epidemiological characteristics, diagnostic features, and treatment. We also aim to highlight the important and recommended preventative strategies and future directives for management, including novel biomarkers and precision medicine and to present a simplified approach to diagnosis, compiling all the recent evidence/guidelines. We believe our study could help create awareness and also help in planning future trials on hepatitis of unknown origin. In our article, we have used the term “acute non-HepA–E hepatitis” to refer to hepatitis of unknown etiology/origin in children as described by the WHO and the UK Health Security Agency [5,20] (Figure 1).

## 2. Acute Non-HepA–E Hepatitis

**Figure 2:**
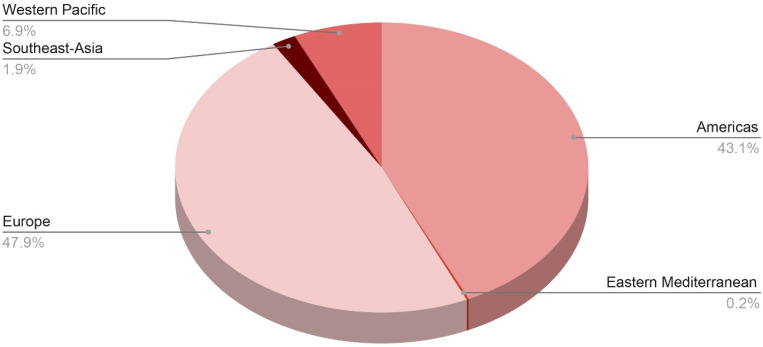
Distribution of probable cases of severe acute hepatitis of unknown origin across the WHO regions.

Since October 2021, there have been several clusters and lone instances of acute hepatitis reported in the US, Europe, and most recently in Asia and Central America. The term “acute non-HepA–E hepatitis” has been coined after laboratory analysis of the common viral hepatitis agents (HAV, HBV, HCV, HDV, and HEV) produced negative results. The precise underlying etiology has yet to be determined; however, one leading idea is that an infectious agent is the culprit for the incidence of acute non-HepA–E hepatitis. So far, laboratory testing has revealed the presence of group F human adenovirus serotype 41 (HAdV-F41) in around three-fourths of the cases studied. Figure 2 shows distribution of probable cases across WHO regions. More than 450 cases had been reported worldwide as of 13 May 2022, with the majority occurring in the United Kingdom (n = 176), the United States (n = 109), 13 European countries (at least 103 cases), and Argentina, Brazil, Canada, Costa Rica, Indonesia, Israel, Japan, Palestine, Panama, Singapore, and South Korea [15].

As of 8 July 2022, the WHO European Region has reported 484 probable cases from 21 countries, including 272 cases (or 27% of all cases worldwide) from the United Kingdom of Great Britain and Northern Ireland (the UK), whereas the region of the Americas has reported 435 cases, including 334 cases (33% of global cases) from the United States of America. Following that is the Western Pacific region with 70 cases, the South-East Asian region with 19 cases, and the Eastern Mediterranean region with only 2 cases [21].

Other countries that have reported the disease include: In Europe: Spain (40), Italy (36), Portugal (19), Ireland (17), Netherlands (15), Belgium (14), Sweden and Greece (12), Poland (11), Denmark and France (8), Israel and Norway (5), Austria (3), Cyprus (2), Bulgaria, Latvia, Luxembourg, Moldova, and Serbia (1). In the Americas: Mexico (69), Canada (21), Argentina and Costa Rica (3), Brazil and Colombia (2), and Panama (1). In Asia: Japan (67), Indonesia (18), Singapore (3), and the Maldives (1). In the Eastern Mediterranean: occupied Palestinian territories and Qatar (1) until now, with an expectation for case count change after the verified data update [21].

The clustering of cases of acute non-A–E hepatitis from Scotland since 31 March 2022 has invited extra attention owing to the high risk of liver failures [22]. This acute hepatitis, often accompanied by multi-system inflammatory syndrome (MIS-C) has affected children between the ages of 1 month up to 16 years old, predilected for those aged between one to five years in 364 of 479 cases as of 8 July 2022. The protocol used for reporting has three pillars: the probable case that is 16 years old or younger since the 1 October 2021 and has acute hepatitis (non-hepatitis viruses A, B, C, D, and E) with aspartate transaminase (AST) or alanine transaminase (ALT) higher than 500 IU/L, the Epi-linked case that is of any age presenting with acute hepatitis (non-hepatitis viruses A, B, C, D, and E) and has been in contact with a probable case since 1 October 2021, and the discarded case who was classified as a case but who, upon additional inquiry, did not fit the requirements for a case. Hepatitis cases with recognized etiologies, such as those caused by specified infectious diseases, medication toxicity, metabolic genetic conditions, or autoimmune diseases, should not be reported using this approach. The approach to defining hepatitis in current focus has evolved over time (Figure 1) [3].

Admitted acute non-A–E hepatitis patients are assigned to specific diagnostic codes contained within the International Statistical Classification of Diseases and Related Health Problems 10th Revision (ICD-10). The codes include K759 for unspecified inflammatory liver diseases, K752 for nonspecific reactive hepatitis, K720 for acute and subacute hepatic failure, K716 for toxic liver disease with hepatitis, which is not elsewhere classified, B190 for unspecified viral hepatitis with hepatic coma, B199 for unspecified viral hepatitis without hepatic coma, B179 for unspecified acute viral hepatitis, and B178 for other specified acute viral hepatitis [23].

## 3. Clinical and Socio-Epidemiological Characteristics of Non-HepA–E Hepatitis

Acute non-HepA–E hepatitis primarily affects previously healthy children aged 16 years or younger with no comorbidities. The median age is three years in both the United States and Scotland. The majority of the affected children were 10 years old or younger. Specifically, 12 of the 13 cases reported in Scotland involved children aged 5 years or younger. Six of the nine patients in Alabama, United States were under the age of five. The most common symptoms reported preceding the hospitalizations were abdominal pain, vomiting, and diarrhea. Furthermore, very high levels of liver enzymes (ALT and AST) have been observed in conjunction with jaundice. Very high levels of serum aminotransferases, exceeding 500 IU/L, are a striking feature [15]. The majority of children who developed acute hepatitis of unknown origin were previously healthy children with no chronic disease. Consequently, hereditary metabolic diseases or genetic abnormalities should be ruled out. The most common presenting symptoms were gastrointestinal symptoms, which were often accompanied by respiratory symptoms reported in the weeks preceding hospital admission. The majority of patients sought medical attention after developing jaundice (yellowing of the eyes or skin, dark urine) [24]. Some patients had their hepatic histopathology evaluated. In the United Kingdom, liver specimens included eight biopsies and six liver tissue samples obtained from children undergoing liver transplantation. The histopathologic examination revealed a range of severity ranging from mild hepatocellular injury to massive hepatic necrosis. Non-specific changes were seen in the histopathologic pattern [25]. The majority of the affected children had abnormal liver function tests (LFTs), with a hepatocellular pattern and serum alanine aminotransferase (ALT) or aspartate aminotransferase (AST) elevations greater than 500 U/L, with a subset, also having bilirubin elevation. This pattern is consistent with virally mediated hepatocyte injury seen in children with hepatotropic and non-hepatotropic viral etiology, but without corresponding markers for immune-mediated, genetic, or metabolic liver injury. However, the case definition criteria are based on 500 IU/L of ALT or AST, but the values reported are not exhaustive. Serum ALT ranged from 603 to 4696 IU/L (median: 1724 IU/L), serum AST ranged from 447 to 4000 IU/L (median: 1963 IU/L), and total bilirubin (TB) levels ranged between 0.23 and 13.5 mg/dL. 12. This suggests that the hepatocytes of the affected children were severely damaged [24].

Over a hundred children have been found to have an unusual form of hepatitis with negative serology for Hep A–E. The majority of cases are in the United Kingdom, the United States, Spain, and Ireland; however, there may be a thousand or more affected patients. Patients include mainly infants between the ages of 1 and 5 years, but teenagers and adults have also been documented. Some patients require liver transplantation. In contrast to the United States, where the states of Alabama and North Carolina first saw the majority of cases, the majority of cases in the United Kingdom were in England. A viral origin has been hypothesized in some cases. Other studies have shown signs of either an autoimmune etiology or a metabolic-genetic basis. On 15 April 2022, the World Health Organization (WHO) stated, that it is necessary to determine the cause of these occurrences in order to advance clinical and public health actions [6].

Only three of the cases had a history of close contact and most of the cases occurred randomly in various nations or regions without any clear epidemiological associations. No presence of any identifiable exposures, such as to particular chemicals, medications, food, or water, or a history of travel to the epidemic area have been noted. Remarkably, few of the afflicted children had a background in SARS-CoV-2 or (COVID-19) immunization.

## 4. Current Understanding of the Etiopathogenesis

The exact pathogenesis is not well elucidated, although there are multiple hypotheses explaining it. In the second technical briefing, the UKHSA hypothesized that the underlying cause can be an adenovirus infection acting over abnormal susceptibility or host response; priming by prior COVID-19-related infection; mutation leading to a novel variant adenovirus; or co-infection with SARS-CoV-2. They also suggested that a novel undiscovered virus; a drug, toxin, or environmental exposure; a new variant of SARS-CoV-2 could play a role as well [23]. However, we strongly believe that it is not caused by traveling (clusters of cases are not seen) [26].

### 4.1. Adenovirus

Adenovirus (AdV) has been the most commonly isolated organism in all cases [8] and is a major focus of the investigation after the WHO stated around 70% of cases show its presence. Recently, the Joint ECDC and the WHO Regional Office reported 53.1% of cases positive for adenovirus in the European region. The deficient immunity caused by reduced exposure to pathogens during the COVID-19 pandemic may have rendered them susceptible to severe forms of infection from common pathogens (e.g., adenovirus) [27,28]. Amongst 260 UK cases, 241 have been tested for adenovirus, of which 156 (64.7%) had adenovirus detected [29]. The CDC tested 299 patients for adenovirus and found 49% positive results, with viral typing done in 22 patients. Hexon gene sequencing confirmed the following: 13 cases were type 41, 1 case was type 40, and 6 cases were other adenovirus types. The CDC identified that tests using whole blood were more sensitive than those using plasma. However, they also found no typical adenovirus findings in the hepatocytes: viral inclusions, negative immunohistochemistry, and electron microscopy, though the biopsies revealed varying degrees of active hepatitis [7]. Baker and colleagues had a similar finding in the liver biopsy samples in a series of 9 cases having a similar presentation in Alabama [2]. Adenovirus type 41 spreads mostly through the fecal–oral pathway and primarily affects the gut. It is a common cause of pediatric acute gastroenteritis, which is characterized by diarrhea, vomiting, and fever, and is frequently accompanied by respiratory symptoms. Adenovirus is known to cause hepatitis in immunocompromised children. There have been various speculations on molecular mechanisms behind adenoviral acute hepatitis in children, including but not limited to the role penton protein of virus, immunological hypothesis, and direct viral injury. It may be an underappreciated cause to liver injury in healthy children; however, the exact understanding of these mechanisms and this association awaits more work to reach plausible evidence [2,23,28]. On 17 August 2022, the CDC also reported that incidence is at baseline pre-pandemic levels and is not a cause for concern [7].

### 4.2. COVID-19 Infection (SARS-CoV-2)

Acute hepatitis can be a sequela of SARS-CoV-2 infection. SARS-CoV-2 has been detected in 15.2% of the tested cases from the UK [8]. Interestingly, almost all cases from Israel have a positive current or past COVID-19 infection [30]. COVID-19-associated hepatitis in Children (CAH-C) was reported in 37 children after having an “asymptomatic” COVID-19 infection. Their inflammatory markers were not elevated and these patients achieved full recovery with supportive treatment. Li-Ya Zhang et al. noticed that in the Chinese population, the omicron variant of COVID-19 caused many GI symptoms. The ACE2 receptor—through which SARS-CoV-2 gains entry—is present in most cells in the body, including the liver. Yet most liver derangements peri-COVID infection have been mild or transient. However, the risk increased if the patient had pre-existing liver disease. Furthermore, the drugs used to treat COVID-19 infection have been implicated in this derangement and studies need to be done to clarify the hypothesis. Severe jaundice and cholangitis have been rare findings. Therefore, they suggest that a direct association between COVID-19 infection and acute non-HepA–E seems unlikely [24,31]. A series of cases of children in Alabama who had a similar clinical picture of hepatitis also had negative tests for current or past COVID-19 infection [2,7]. Petter Brodin and colleagues described that the SARS-CoV-2 spike protein can elicit a cytokine storm akin to Toxic Shock Syndrome (TSS) superantigen, since they have a similar motif. Li-Ya Zhang et al. suggest that adenovirus infection in the background of an intestinal viral reservoir of SARS-CoV2 can become activated, triggering immune-mediated hepatitis [24,32]. The COVID-19 vaccination was linked to causing acute non-HepA–E but was rejected since most of the infected children were not vaccinated [33].

### 4.3. Other Viruses

Adeno-Associated Virus 2 (AAV2) is a dependoparvovirus and requires co-infection of other viruses—adenovirus or herpesvirus (HHV)—to replicate. It generally does not cause any major symptoms, or just causes mild symptoms. Because of its ineffective replicative and infective potential, it has been engineered as a vector for gene therapy [34]. However, Antonia Ho and colleagues isolated this virus in the liver and plasma of all patients in their study. AAV2 was not found in any controls. The controls were formed from a cohort of age-matched individuals infected with adenovirus that have a normal hepatic function and those admitted for hepatitis of other etiology. The lab reports of affected patients showed infection with either HHV-6 or adenovirus. The class II HLA-DRB1*04:01 allele was found in 89% of the cases, whereas the population frequency was seen to be 15.6%. This suggests a possibility of a genetic basis of susceptibility [35].

Morfopoulou et al. identified that AAV2 was seen in the explanted liver in all five patients who underwent liver transplantation and from the blood in 10/11 non-transplanted cases in high levels. AdV and HHV-6B were found in all five explanted livers and the blood from 15/17 and 6/9, respectively, of the 23 non-transplant cases. In the controls, AAV2 was found in only 6/100 controls in low levels in blood samples. The researchers were unable to find a reason to believe that the hepatic pathology was caused by infection by either AdV or AAV2 after attempting to find evidence through electron microscopy, immunohistochemistry, or proteomics of AdV or AAV2 viral particles or proteins [36].

Prior to this outbreak, Patterson and colleagues identified that the most common cause of liver failure is HAV, HBV, and HEV [37]. However, the recent outbreak seems to add a different outlook. The current outbreak has resulted in many pediatric cases facing acute liver failure. Though the above-cited causes form the gist of major attributable evidence, the other implicated etiological agents include human herpes virus (HHV) 7, HHV6, Epstein-Barr virus, cytomegalovirus, respiratory syncytial virus, enterovirus, and influenza A virus. They are now being seen as other probable causative agents, but no concrete evidence has supported this hypothesis. With the evidence available, adenovirus, SARS-CoV-2 virus, or AAV have been seen as the prime infective etiopathological agents.

### 4.4. Non-Infectious Causes

A considerable number of drugs, heavy metals, and toxins cause hepatotoxicity: drugs—amoxicillin-clavulanate, sulfamethoxazole-trimethoprim, ciprofloxacin, isoniazid (INH), NSAIDs, paracetamol; heavy metals—lead, chromium, arsenic, mercury, nickel and cadmium; and toxins—mycotoxins and Garcinia Cambogia are notorious for causing hepatotoxicity [38,39,40,41]. A preliminary investigation linking toxins and acute non-HepA–E revealed no significant findings. The UKHSA tested the samples for paracetamol and fluconazole but found no significant results; they tested for metals and compared it with publicly available data from different regions (USA and Canada), but found no significant association; they are currently attempting to devise a method to identify previous exposure to mycotoxins since most mycotoxins (or any toxins) would have been metabolized and excreted prior to sample collection [8]. The European Society of Clinical Microbiology and Infectious Diseases (ESCMID) also reported the possibility of aflatoxin—which is a mycotoxin known to cause hepatic damage and hepatocellular carcinoma [42]—being involved in pathogenesis [43].

## 5. Approach to Diagnosis

**Figure 3:**
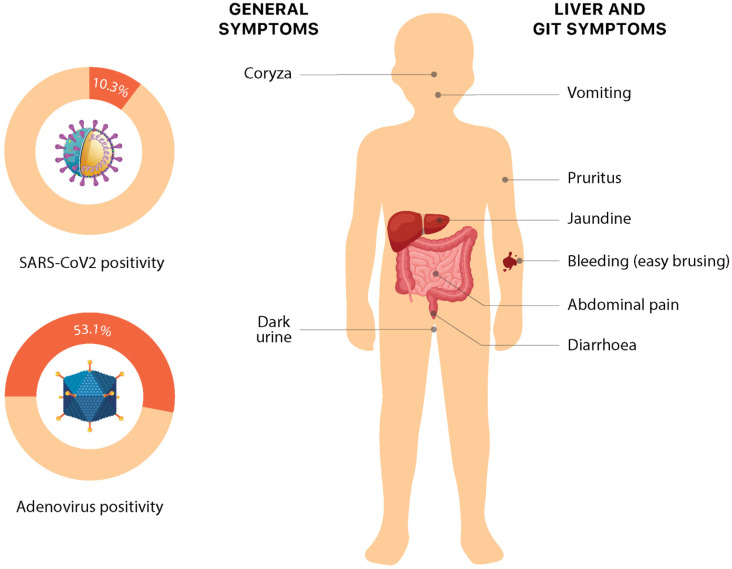
Symptoms and etiology of non-Hep A–E hepatitis. Numeric data is based on the latest available data from the European Region on 30 September 2022 [3].

Recent times have seen a clustering of cases of hepatitis of unknown origin, especially in the pediatric age group. The cases do resemble any other case of acute hepatitis, with the most common symptoms being: jaundice (68.8%), vomiting (57.6%), gastrointestinal symptoms such as abdominal pain (36.1%) and nausea (25.7%). Some non-specific symptoms such as lethargy (48.6%), diarrhea (43.1%), fever (28.5%), respiratory symptoms (18.1%), dark urine (6%), coryza (6%), easy bleeding/bruising, and pruritis (1%) have also been reported (Figure 3). Interestingly, these nonspecific symptoms in these cases have shown a temporal association with liver injury, with the respiratory and gastrointestinal symptoms mostly preceding the onset of jaundice by several weeks. However, current evidence for this association is preliminary and requires more data to make conclusive comments [12,24,33,44]. The symptoms somehow overlap with the presentation of pediatric acute liver failure (PALF) [44,45]. PALF can be precipitated by a variety of causes, including but not limited to drugs, toxins, metabolic and genetic alterations, infections, autoimmunity, hemodynamic disturbances, and oncologic injuries, which vary in congruence to the environmental and socio-epidemiological factors. The major signs upon presentation include scleral icterus, hepatomegaly, ascites, and peripheral edema [46]. Splenomegaly is very rare; however, signs of hepatic encephalopathy may be seen [2].

A characteristic finding has been abnormal liver function tests (LFTs), having a predominant hepatocellular pattern and serum alanine aminotransferase (ALT) or aspartate aminotransferase (AST) elevations greater than 500 U/L, with a small subset having bilirubin elevation. The current guidelines recommend that INR levels equal to or more than 1.5., consistently rising INR or bilirubin levels, and/or new onset of symptoms of liver failure must be taken as red flags that invite immediate action [24,46]. In the cases reported from Alabama, serum ALT levels ranged from 603 to 4696 IU/L (median: 1724 IU/L), serum AST ranged from 447 to 4000 IU/L (median: 1963 IU/L), and total bilirubin (TB) ranged from 0.23 to 13.5 mg/dL [12]. Although the recent literature misses many reports, routine blood tests including those for C-reactive protein, inflammatory markers, immunoglobulin G, and other indications may aid in determining the cause of infection, inflammation, and the immunological state. The WHO has urged that all patients fitting the case definition should have all available samples tested to help detailed diagnosis [24]. When a likely or epi-linked case is detected, a panel of testing has been recommended to identify the pathogen. Blood should be examined for adenoviruses, enteroviruses, CMV, EBV, HSV, HHV6, HHV7, and parechoviruses. Throat swab specimens should be examined for respiratory viruses (including influenza, adenovirus, parainfluenza, rhinovirus, respiratory syncytial virus, and human bocavirus 1–3), SARS-CoV-2, enteroviruses, and human metapneumovirus (hMPV). Stool samples should be examined for enteric viruses (such as norovirus, enteroviruses, rotavirus, astrovirus, and sapovirus). In addition, the ECDC recommends testing for Brucella spp., Bartonella henselae, and Borrelia burgdorferi serology (if epidemiologically relevant). The culture of bacterial pathogens and viruses is another important and useful tool. Metagenomic analysis (blood and liver specimens) can also be done [12].

With the investigations, imaging has always proven to be a much-needed extension. In congruence to that for the recent cluster, abdominal USG proved to be a very useful extension to clinical examination and helped elucidate many characteristic findings, including gall bladder thickening (20%), hepatomegaly (12%), splenomegaly (8%), abdominal lymph nodes (6%), abdominal fluid (1%), and “Starry sky” appearance (accentuated portal venules with diminished liver parenchymal echogenicity) (2%) [46]. The other more refined imaging assets can be CT and MRI, which can help identify mild hepatomegaly, periportal edema (decreased attenuation around the portal system and at the hepatic hilum on CT or increased T2 signal around the portal system on MRI), and periportal lymphadenopathy. An electroencephalogram may be employed for patients with suspected hepatic encephalopathy. A head computed tomography (CT) or MRI can be used to rule out cerebral edema and cerebrovascular accidents [47].

Although liver biopsy is not routinely performed, it is indicated that when the diagnosis is unclear, such as in patients with atypical clinical features, no-response to treatment, co-existing chronic liver disease (congenital metabolic disease in children), inconclusive extensive biochemical workup, fever of unknown origin, and equivocal findings on imaging studies have been observed. Percutaneous, CT/US-guided, transjugular (transvenous), and Endoscopic Ultrasound (EUS)-guided biopsies are the most regularly used procedures for liver biopsies [48,49].

It has been observed that early diagnosis helps improve the prognosis largely for any disease, including hepatitis. In a situation of clustering of cases, risk stratification becomes imperative. With the enhanced and informed application of clinical skills with point- of-care tests and portable imaging, the “screen-at-the-clinic” approach can be taken and it can help not only by reducing morbidity but also DALY caused by the disease.

With the clustering of diseases, public health response and planning depends on establishing a clear working definition, and the WHO has presented a working case definition of confirmed, probable, and epi-linked cases: (1) Confirmed: Not applicable at present; (2) Probable: A person presenting with acute hepatitis (non-HepA–E) with serum transaminase >500 IU/L (AST or ALT), who is 16 years and younger, since 1 October 2021; (3) Epi-linked: A person presenting with acute hepatitis (non-HepA–E) of any age who is a close contact of a probable case, since 1 October 2021. It has clearly laid out that if hepatitis A–E serology results are pending and other criteria met, the cases should be reported as “pending classification”. However, they have recommended discarding cases with other explanations for their clinical presentation [33].

**Figure 4:**
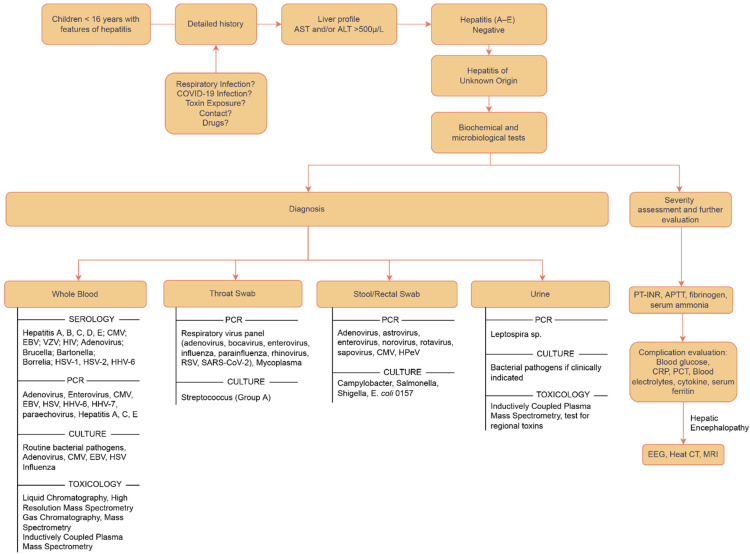
Flowchart depicting the approach to diagnosis for acute hepatitis of unknown origin [47,50].

Various attempts have been made to propose an organized approach to diagnosis. The core remains that the basic approach is to identify all susceptible cases presenting with symptoms of acute hepatitis and screen them for Hep A–E; if negative, a detailed systematic investigation and evaluation must be carried out in the best capacity to reach the conclusion in formulating a diagnosis and planning the treatment. All possible differentials need to be catered to one by one, starting with the antigenic point-of-care tests using blood, urine, and stool samples, and then shifting to biochemical, microbiological investigations and imaging, and then exploring causes relating to toxins, genetics, and environmental antigens [47]. The order can be individualized as per the patient’s history and examination. A sample approach has been outlined in Figure 4.

## 6. Treatments

The treatment of acute hepatitis relies largely on identifying the etiology and targeting the pathology. This makes the treatment of acute non-HepA–E hepatitis rather challenging because of our limited understanding of its etiopathogenesis. Therefore, the management of acute hepatitis of unknown origin should be based on the coverage of theorized etiologies. The mainstay of treatment of acute hepatitis of unknown origin is supportive therapy and management of all the complications of hepatocellular insufficiency [33]. A few targeted therapies based on possible etiologies and hepatoprotective effects may have a role in improving the prognosis, promoting resolution, and reducing the need for liver transplantation [33,51].

### 6.1. Symptomatic and Supportive Therapy

Supportive therapy is the cornerstone of the management of acute hepatitis of unknown origin. The general measures include adequate rest, maintaining optimal hydration and electrolyte balance, and regular monitoring of liver function test (LFT), prothrombin time (PT), and volume status. In addition to preventing progression to acute liver failure, the goal of supportive therapy is to prevent serious complications associated with hepatocellular dysfunction including coagulopathy, hepatorenal syndrome, hyperbilirubinemia, and hepatic encephalopathy [47,51,52].

In patients presenting with acute liver failure, fluid resuscitation along with the management of complications is appropriate [52,53]. In hemodynamically unstable patients with a hypoalbuminemic milieu, fluid resuscitation should be exercised with utmost caution to prevent fluid overload and precipitation of pleural effusion, ascites, or cerebral edema. Optimal hydration should be maintained to prevent shock and hepatorenal syndrome [52].

### 6.2. Specific/Targeted Therapy

The precise therapy varies as per the causative agent and severity of the disease. The clinicians must attempt to individualize the treatment as per the patient.

#### 6.2.1. Anti-Viral Drugs

##### Drugs against Human Adenoviruses

Adenovirus is commonly hypothesized to be involved in acute non-HepA–E hepatitis. Although there is no approved therapy for adenovirus, Cidofovir and Ribavirin have shown improvement in transplant patients [54,55]. Cidofovir is a cytosine analog that suppresses viral DNA replication by insertion into growing DNA and inhibiting it. It is well tolerated and thus the drug of choice for disseminated adenovirus infections [56]. The evidence of Ribavirin’s being effective against adenovirus has been scarce and more primary evidence is necessary to prove its effectiveness [55]. It is a guanine nucleotide analog that works on various DNA/RNA viruses by halting viral replication and inducing mutation in the viral genome. It is presumed to be severely teratogenic and can cause hemolytic anemia.

##### Drugs against COVID-19

A number of drugs have been FDA approved for use for COVID-19 infection, but none has yet been identified or found emergency approval for cases of hepatitis. These require more work. Furthermore, the safety profile of these new or repurposed antivirals in children (prime target of the outbreak) still remains a question. In addition, in view of the doubtful etiology of SARS-CoV-2 in the current outbreak of hepatitis, a COVID-specific drug may not be what we are looking for unless strongly indicated for an infection confirmed by a RTPCR test.

#### 6.2.2. Glucocorticoids

Inflammation due to non-infectious etiologies in acute non-HepA–E hepatitis (auto-immune hepatitis) could benefit from the anti-inflammatory profile of glucocorticoids. Thus, glucocorticoids could be useful in many patients and may be tried in severe cases. If the risk of aggravation of infectious agents is more likely, then it should be assessed before initiating glucocorticoids. Ultimately, it is up to the provider’s discretion if they want to initiate glucocorticoid therapy for the patient [25,57].

#### 6.2.3. Plasmapheresis

Plasmapheresis is touted to be effective in adults with acute liver failure (ALF) or coagulopathy, but the evidence in children is limited. Plasmapheresis may slow down the disease course but has not been shown to improve mortality or outcomes [52].

### 6.3. Hepatoprotective Drugs and Phytochemicals

Supplementing the supportive and targeted therapy with herbal drugs and phytochemicals possessing antioxidant and hepatoprotective properties has been explored. Numerous phytochemicals—found in cranberry, aloe vera, beetroot, artichokes, spirulina, and turmeric—are believed to have hepatoprotective and antioxidant properties which could improve outcomes in acute non-HepA–E hepatitis and prevent progression to ALF [58].

### 6.4. Management of Complications

#### 6.4.1. Coagulation Disorders

One of the major complications of acute non-HepA–E hepatitis is the progression of hepatitis to ALF. This results in the failure of endogenous synthetic, metabolic, and excretory functions of hepatocytes, leading to several complications. One of the primary synthetic functions of the liver is coagulation factors synthesis. Thus, without coagulation factors, an elevated PT and PT-INR ensue, a hallmark of hepatocellular failure. Moreover, decreased anticoagulants, decreased thrombopoietin, and systemic inflammation may contribute to a complex coagulopathy in ALF [47]. Although the elevated PT-INR points towards the increased hemorrhagic tendency in these patients, there is ample evidence indicating that there is hypercoagulability as well. Therefore, homeostasis in ALF is a rebalanced state. Once coagulopathy develops, regular monitoring of the PT-INR is imperative to manage it appropriately. Because it is a complex, rebalanced state, and the mortality benefit of blood transfusion is unclear, our goal must be to treat the underlying issue before it progresses to this stage. [59]. Plasma infusion may improve PT but always carries a risk of volume overload [52]. An appropriate dose of Vitamin K should be administered initially to correct the coagulopathy [47,52,60].

#### 6.4.2. Hepatic Encephalopathy

Hepatic encephalopathy can develop due to inadequate ammonia handling by the failing liver. Assessment of hepatic encephalopathy should be carried out by physical examination as well as venous ammonia monitoring. Ammonia reduction therapies through oral lactulose or rifaximin should be initiated. Lactulose traps ammonia in the intestinal lumen by acidifying the lumen and promoting the excretion of ammonia. Rifaximin reduces the ammonia-producing bacteria in the intestinal lumen [47,52,60].

#### 6.4.3. Intracranial Hypertension (ICH)

Intracranial hypertension in ALF develops due to increased cerebral blood flow and systemic inflammation and contributes greatly to mortality in these patients. It can be managed with the help of IV mannitol, furosemide, and hypertonic saline [61].

#### 6.4.4. Acute Liver Failure

Extracorporeal liver support systems: ALF precipitated by fulminant hepatitis, artificial liver support (ALS), and Bioartificial Liver support (BLS) systems may be tried before a donor is available. It has demonstrated temporary efficacy, but survival benefits are unclear.

#### 6.4.5. Terminal Treatment

Acute liver failure (ALF) is defined as a fast decline of liver function (international normalized ratio 1.5) and the development of hepatic encephalopathy in a patient with no prior history of liver illness within 26 weeks after jaundice [62]. ALF accounts for 8% of liver transplantation (LT) indications in Europe and 7% in the United States [63]. Patients with dire prognoses should be considered for a timely liver transplant. High mortality and graft loss rates endure despite continuous improvement in ALF survival after LT, particularly in the first three months following transplant. However, these values need to be fine-tuned to better identify patients who will benefit from transplantation. Current prognostic models are useful in identifying people who will need LT. To increase survivability, better prognostic strategies and technologies are under development [64].

## 7. Prevention

The idiopathic etiology of “Acute hepatitis of unknown origin” makes it hard to establish definite preventative guidelines. Considering that some cases required liver transplantation, the impact of this disease on children cannot be overlooked. Therefore, researchers are constantly trying to piece together the puzzles from prior cases to establish preventative guidelines. Furthermore, many cases were related to patients who were reported to be otherwise immunocompetent and healthy before the episode with this disease. Therefore, it is recommended that precautions be taken seriously by everyone to prevent disease contraction, especially children aged 16 years and below.

So far, the researchers have been unable to identify a common exposure to the environment, water, food, medicine, or any association with parents’ occupation or travel history [25]. However, the histopathology of this disease can be used to our advantage. Some non-hepatotropic viruses such as adenovirus (53%), Human herpesvirus 7 (33%), Enterovirus (22%), Human herpesvirus 6 (16.6%), Epstein–Barr virus (15%), SARS-CoV-2 (10.3%), and some others are being considered as causative agents. Researchers can use the modes of transmission and preventative measures of these viruses and apply them to acute hepatitis of unknown origin (Joint ECDC-WHO Regional Office for Europe Hepatitis of Unknown Origin in Children Surveillance Bulletin). For example, adenovirus transmits via fecal–oral and respiratory routes. Therefore, both “contact” and “droplet” precautions are adopted against it (Table 1). Fecal–oral transmission can be prevented by adopting good hygiene practices which entail washing hands with water and soap for at least 20 s. Children should avoid touching their faces, eyes, mouth, and nose with unwashed hands [65]. Sick children should stay at home to minimize contact and protect others. They should avoid personal contact such as touching, shaking hands, or sharing utensils with others. They should cover their mouth with a tissue or some cloth when coughing or sneezing. It is encouraged to clean and disinfect surfaces frequently to eliminate lingering virus particles. Adenovirus tends to be resistant to many common disinfectants; therefore, the CDC recommends using EPA-registered disinfectants. The respiratory route can be avoided by practicing droplet precautions and social distancing. Generally, if the aerosolized particles are ≥5 microns, they cannot stay in the air for too long and only transmit 3–6 feet. Therefore, droplet precautions (e.g., surgical masks) can be used when within the range of infection. The CDC also recommends that healthcare personnel are educated on the importance of source control to prevent viral transmission by adequately containing respiratory secretions, especially during seasonal outbreaks of viral respiratory tract infections. Moreover, the virus can also be shed into the feces of infected people and spread to others. Therefore, it is important to keep water supplies clean. Free chlorine has been shown to be effective against adenovirus [66].

In addition to personal protective equipment and transmission protocols, vaccines are another popular preventative tool employed against several infectious diseases. Because of the unascertained cause of acute hepatitis of unknown origin, setting the vaccination guidelines or steering the vaccine development in the right direction is challenging. Despite this obscurity, the current evidence about the hypothesized infectious agents directs us to use the pre-existing vaccines against these agents to add to the layer of protection and decrease the likelihood of contracting these agents. There are live oral vaccines against AdV infection that reduce the risk of respiratory infection. However, these vaccines are currently only for military use and are not available for civilians [65]. The focus right now is on finding the causative agent. Once that is established, researchers can look into repurposing the current vaccines by using currently available vaccines. Repurposing is more cost-efficient and less time-consuming than developing the vaccine from scratch.

Once an outbreak occurs or is on the brink of occurring, the most effective tool is a timely and efficient reporting system. A secured global information center or a sample bank with information about all cases of the diseases is crucial to establishing the cause and treatments. WHO launched a global online survey on 11 July 2022, to estimate the incidence and other relevant information about the disease. Further investigations are being conducted by national authorities to gain more insight into any microbiological or toxicological testing. A recent study by Branda et al. has also emphasized the need for an “open access” database to provide reliable unbiased clinical data from around the globe, aiding in real-time situation analysis, better coordination, early diagnosis, timely containment, enhanced public health information, and improved decision making by policymakers [67].

It takes collective efforts from global and national authorities to prevent an outbreak from becoming an epidemic or pandemic. The reporting and sharing of correct evidence-based information become imperative. The collection of evidence-based data about acute hepatitis of unknown origin and making it accessible can facilitate researchers’ employing this data to investigate the causes, associations, and risk factors. This is key to developing robust treatment plans and preventative guidelines.

## 8. Future Perspectives and Way Ahead

The recent outbreaks over the past three years have helped us direct our attention to the need for better pandemic preparedness protocols. In the era of prevention and precision medicine, we were somehow waiting for an eye-opener to integrate precise preventive strategies into our healthcare system. Although we have reporting systems for diseases following national and international protocols, the agencies still need to refine them and produce elaborate guidelines that are regularly updated by global consensus. We still wait to see a time when the vaccination and drugs can be guided and based on data covering the “omics” and preventive strategies that can be geographically and socio-epidemiologically crafted. At the level of a clinician, a “screen-at-clinic” approach can be undertaken, taking into consideration the geographical and temporal prevalence. The integral step to an imagined future is seeding an “independent” reporting system supported by a “sample-bank”, allowing for a global collaboration on public data. If we can reach a state where we have genomic, metabolomic, proteomic, and immunological data representative of the human race, we will be able to test it against data from samples derived from each cluster of diseases that appears and the age of human dominance and in-silico trials can be thought of. With the advent of AI and organ-on-a-chip (OOC) to study hepatitis, the landscape of modern medicine may look overtly futuristic unless we find robust evidence about the practicality of these interventions, but with the direction and speed of current developments, there is hope to enhance the current approach [68].

## 9. Conclusions

The crux of all discussions about acute hepatitis in children is that still it has no identified cause. The current evidence strongly suggests the role of infectious agents such as adenovirus or the severe acute respiratory syndrome coronavirus 2 (SARS-CoV-2). Other pathogens associated with acute hepatitis of unknown origin are AAV2, HHV6, HHV7, and human polyomavirus, but the relevant evidence is still under investigation. The literature also points to the possibility of children with acute severe hepatitis having underlying immunodeficiencies, but we need more insight to understand this disease from an immunological perspective. Although the clinical characteristics and the unusually high prevalence of adenoviruses among the reported cases suggest that infectious agents play a more important role in acute non-HepA–E hepatitis, other possible etiologies such as environmental agents, toxins, or foodborne etiologies should be thoroughly investigated.

Precautions need to be planned in countries with no reported cases and those with higher prevalence. Clinicians should get acquainted with the terms and etiologies to deal with this severe unidentified form of hepatitis. To generate public awareness and educate patients about this entity, utmost care should be taken to not create panic yet equip them to prevent the occurrence. We advocate that precise preventive measures be planned and executed at both private and public health facilities to contain the outbreak and prevent its recurrence.

## Figures and Tables

**Table 1:** Preventive strategies for hepatitis of unknown origin.

Serial No.	Prevention Strategies
Both contact and droplet precautions are required to prevent the spread of causative agents.	
1.	Wash hands with water and soap for at least 20 s.
2.	Avoid touching face, eyes, mouth, and nose with unwashed hands.
3.	Stay at home or minimize contact if sick.
4.	Cover mouth with a cloth when coughing or sneezing.
5.	Social distancing.
6.	Water supplies should be kept clean (free chlorine can be used).
7.	Clean surfaces using standard disinfectants.
8.	Healthcare personnel should be educated on the importance of source control.
9.	Well-planned usage and repurposing of the available vaccines.
10.	Timely and efficient reporting systems at loco-regional, national, and international levels.

## Data Availability

All the data available on this manuscript.
